# A Cross-Sectional Study of Protein Changes Associated with Dementia in Non-Obese Weight Matched Women with and without Polycystic Ovary Syndrome

**DOI:** 10.3390/ijms25042409

**Published:** 2024-02-18

**Authors:** Alexandra E. Butler, Abu Saleh Md Moin, Thozhukat Sathyapalan, Stephen L. Atkin

**Affiliations:** 1 Research Department, Royal College of Surgeons of Ireland, Busaiteen P.O. Box 15503, Bahrain; amoin@rcsi.com (A.S.M.M.); satkin@rcsi.com (S.L.A.); 2 Academic Endocrinology, Diabetes and Metabolism, Hull York Medical School, Hull HU6 7RU, UK; thozhukat.sathyapalan@hyms.ac.uk

**Keywords:** polycystic ovary syndrome, PCOS, amyloid-associated proteins, Alzheimer’s disease

## Abstract

Dysregulated Alzheimer’s disease (AD)-associated protein expression is reported in polycystic ovary syndrome (PCOS), paralleling the expression reported in type 2 diabetes (T2D). We hypothesized, however, that these proteins would not differ between women with non-obese and non-insulin resistant PCOS compared to matched control subjects. We measured plasma amyloid-related proteins levels (Amyloid-precursor protein (APP), alpha-synuclein (SNCA), amyloid P-component (APCS), Pappalysin (PAPPA), Microtubule-associated protein tau (MAPT), apolipoprotein E (apoE), apoE2, apoE3, apoE4, Serum amyloid A (SAA), Noggin (NOG) and apoA1) in weight and aged-matched non-obese PCOS (*n* = 24) and control (*n* = 24) women. Dementia-related proteins fibronectin (FN), FN1.3, FN1.4, Von Willebrand factor (VWF) and extracellular matrix protein 1 (ECM1) were also measured. Protein levels were determined by Slow Off-rate Modified Aptamer (SOMA)-scan plasma protein measurement. Only APCS differed between groups, being elevated in non-obese PCOS women (*p* = 0.03) relative to the non-obese control women. This differed markedly from the elevated APP, APCS, ApoE, FN, FN1.3, FN1.4 and VWF reported in obese women with PCOS. Non-obese, non-insulin resistant PCOS subjects have a lower AD-associated protein pattern risk profile versus obese insulin resistant PCOS women, and are not dissimilar to non-obese controls, indicating that lifestyle management to maintain optimal body weight could be beneficial to reduce the long-term AD-risk in women with PCOS.

## 1. Introduction

There is an increased prevalence of metabolic features including T2D, hypertension, fatty liver disease and cardiovascular disease [1] in women with polycystic ovary disease (PCOS) that is thought to be related to the degree of insulin resistance and inflammation driven by obesity [1,2], characteristics typically found in the condition. Women with PCOS are reported to suffer more from mood disorders, such as anxiety and depression, and sleep disturbances compared to women without PCOS, indicative of a neurological component to the PCOS disease spectrum [3].

Dementia is predicted to increase markedly as the population ages, potentially rising to 115.4 million by 2050 [4], of which Alzheimer’s disease (AD) accounts for 80% of all cases [5]. Obesity is associated with AD [6], and increased insulin resistance (IR) is also associated with AD independently [7], as insulin is able to freely cross the blood brain barrier [7], and it has been suggested that the underlying molecular mechanisms of IR and AD are due to insulin receptor substrate 1 (IRS-1) and insulin-like growth factor 1 (IGF-1) receptor dysregulation [8]. It has been suggested that the link between obesity, depression and AD is enhanced neuroinflammation [9]. Obesity and increased IR are both characteristic features found in T2D and the increased risk for T2D patients developing AD has been well established [10,11,12]. It has been reported that a pattern of AD-related risk proteins, particularly amyloid precursor proteins (APP), amyloid P component (APCS) and alpha synuclein (SNCA) are found in T2D [13] and recently a similar pattern of these proteins was reported in PCOS [14]. Perhaps this similarity is not surprising as both obesity and increased IR are commonly found in PCOS and it is reported that 10% may develop diabetes [15]. In addition, mood disorders that include both anxiety and depression, and sleep disturbances are more commonly found in women with PCOS compared to those women that do not have PCOS [3]; thus, the combination noted above of obesity and depression with AD [9] may also reflect an enhanced risk of AD in PCOS. Indeed, there is increasing evidence to suggest that there may be a link between Alzheimer’s disease and PCOS [16]. The underlying mechanism has been suggested to be multifactorial with contributions from insulin resistance, obesity and hormonal imbalance from both the pituitary and the ovaries associated with the PCOS condition, all of which can affect cognitive function and increase inflammation [17]. Functional magnetic resonance (MRI) studies have suggested that there are cerebral changes in areas associated with cognition that relate to insulin resistance [18] and to luteinizing hormone (LH) level changes [19], whilst others have suggested that changes in PCOS-related cognitive function are exacerbated by hormonal changes involving increased testosterone [20] and insulin levels [21].

In a proteomic study used to analyze proteins in a comparison between AD, frontotemporal dementia (FTD) and controls, it was reported that five proteins, fibronectin (FN), fibronectin fragment 3 (FN1.3), fibronectin fragment 4 (FN1.4), Von Willebrand factor (VWF) and extracellular matrix protein 1 (ECM1) were discriminatory being increased in AD in comparison to both FTD and controls [22], and which may prove to be important biomarkers for AD in the future. Other proteins central to the biology of amyloid Beta (Aβ), a characteristic pathological feature of AD, include APP, SNCA, APCS, Pappalysin (PAPPA), Microtubule-associated protein tau (MAPT) and apolipoprotein E (apoE) and its alleles (apoE2, E3 and E4) and thus their levels were determined here.

An elevation of APP and APCS, which are associated with AD, and decreased SNCA were found in patients with T2D [13], and this was reflected in a similar pattern of protein expression in obese and insulin resistant subjects with PCOS [14]. Given that obesity and IR are so closely associated with PCOS they are not easily accounted for statistically; therefore, only a study in PCOS of non-obese women without insulin resistance could answer the question of whether the inherent pathophysiology of PCOS infers a greater risk for AD. Thus, we hypothesized that the pattern of AD-related protein changes found in obese women with PCOS [14] would not have been different to matched controls if weight and insulin resistance had been accounted for in the study design; therefore, we analyzed AD-related protein levels in non-obese, non-insulin resistant women with PCOS compared to a matched non-PCOS control population.

## 2. Results

Baseline data for the 24 PCOS subjects and 24 controls are shown in Table 1. The two cohorts were weight and age-matched, and did not have insulin resistance, but subjects with PCOS did have hyperandrogenemia, with increased C-reactive protein (CRP, an inflammatory marker) and anti-Müllerian hormone (AMH).

The results of the Somascan analysis of Alzheimer’s disease-related proteins are shown in Table 2 for the PCOS and control women.

### 2.1. Levels of Alzheimer’s-Related Proteins in PCOS

Only APCS differed between groups, being elevated in non-obese PCOS women (*p* = 0.03) relative to the non-obese control women (Table 1). The levels of other Alzheimer’s-related proteins, namely APP, SNCA, PAPPA, MAPT, apoE, apoE2, apoE3, apoE4, SAA, NOG and apoA1 were comparable between PCOS subjects and controls (Table 2). The dementia-related proteins FN, FN1.3, FN1.4, VWF and ECM1 did not differ between the non-obese non-insulin resistant PCOS and controls.

### 2.2. Correlation Analyses

For the APCS protein that differed between non-obese non-insulin resistant PCOS subjects and control women, correlations with age, BMI, insulin resistance (HOMA-IR), testosterone, C-reactive protein (CRP) and circulating levels of selected inflammatory proteins and protective heat shock proteins (HSPs) (interleukin 6 (IL6), tumor necrosis factor alpha (TNFa), heat shock protein 90 (HSP90AA1, HSP90) and heat shock protein family D protein 1 (HSPD1, HSP60) were determined.

**Figure 1:**
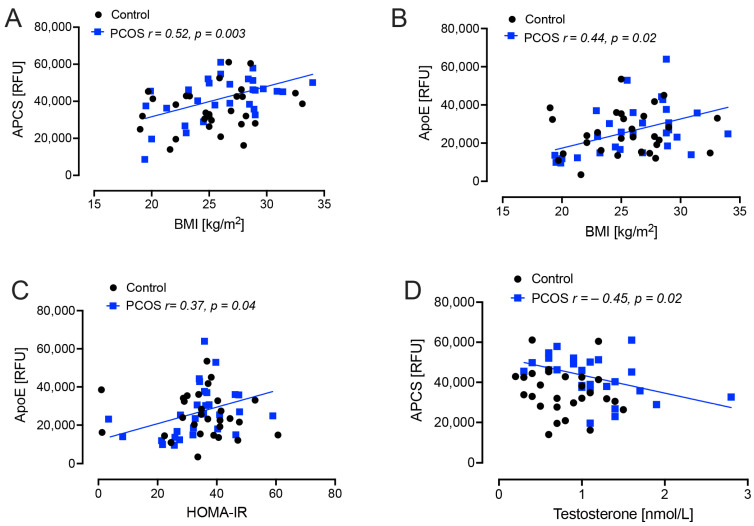
Demographic and biochemical correlations with plasma amyloid-related proteins levels in polycystic ovary syndrome (PCOS) and control subjects; amyloid P-component (APCS) and apolipoprotein E (apoE) with body mass index (BMI), insulin resistance (HOMA-IR) and testosterone in weight and aged-matched non-obese PCOS (*n* = 24) and control (*n* = 24) women. (**A**), positive correlation of APCS with BMI (*p* = 0.003); (**B**), positive correlation of ApoE with BMI (*p* = 0.02); (**C**), positive correlation of ApoE with HOMA-IR (*p* = 0.04); (**D**), negative correlation of APCS with testosterone (*p* = 0.02).

BMI correlated positively with APCS (r = 0.52, *p* = 0.003) and apoE (*r* = 0.44, *p* = 0.02) in PCOS women; Homeostasis model of assessment–insulin resistance (HOMA-IR) correlated positively with apoE (*r* = 0.37, *p* = 0.04) in PCOS women. Testosterone correlated negatively with APCS (*r* = −0.45, *p* = 0.02) in PCOS women (Figure 1).

**Figure 2:**
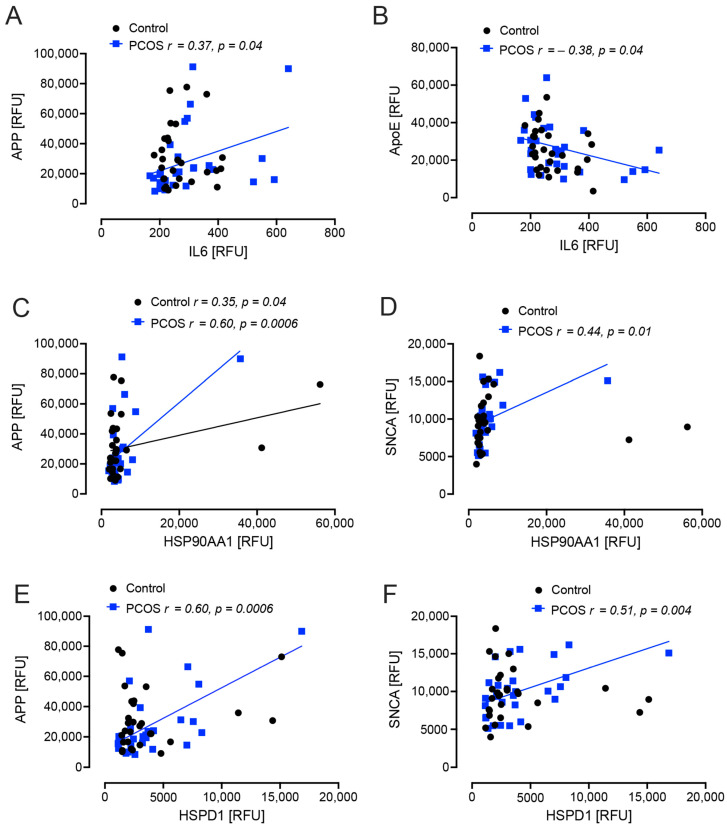
Correlations of Alzheimer’s-related proteins with interleukin 6 (IL6) and heat shock proteins in polycystic ovary syndrome (PCOS) and control subjects. APP correlated positively with IL6 in PCOS (*p* = 0.04) (**A**); ApoE correlated negatively with IL6 in PCOS (*p* = 0.04) (**B**); in both PCOS (*p* = 0.0006) and control women (*p* = 0.04) APP corelated positively with heat shock protein 90 (HSP90AA1) (**C**); SNCA correlated positively with HSP90AA1 (*p* = 0.01) (**D**), APP correlated positively with heat shock protein 60 (HSPD1: *p* = 0.0006) (**E**); SNCA correlated positively with HSPD1 (*p* = 0.004) (**F**). Controls: black open circles; PCOS: blue squares.

Interleukin 6 (IL6) correlated positively with APP (*r* = 0.37, *p* = 0.04) and negatively with apoE (*r* = −0.38, *p* = 0.04) in PCOS women. Heat shock protein 90 (HSP90AA1, HSP90) correlated positively with APP in both control and PCOS women (*r* = 0.35, *p* = 0.04 and *r* = 0.60, *p* = 0.0006, respectively), and correlated positively with SNCA (*r* = 0.44, *p* = 0.01) in PCOS women. Heat shock protein family D protein 1 (HSPD1, HSP60) correlated positively with APP (*r* = 0.60, *p* = 0.0006) and SNCA (*r* = 0.51, *p* = 0.004) in PCOS women (Figure 2).

## 3. Discussion

Here, we show that the only change in plasma Alzheimer’s-related proteins in subjects with PCOS who were non-obese and not insulin resistant was an increase in APCS (*p* = 0.03) relative to weight matched control women. Of note, an increase in APCS was also found in obese women with PCOS [14]. Amyloid P component (APCS) is found in plaques and the neurofibrillary tangles characteristic of Alzheimer’s disease [23], and its role may be involved in the decreased proteolysis of Aβ deposits, leading to further plaque formation [24] therefore, increased serum levels may be detrimental. APCS was shown to accurately discriminate between AD compared to normal brain samples [25]. Overall, the results in this study are in contrast to what has been reported in a cohort of obese women with PCOS from a PCOS biobank [14] where the circulatory AD-related protein pattern reflected what was seen in T2D subjects with elevated APP and lower SNCA [26,27,28]. In this study, the plasma levels of APP, SNCA and apoE were not different between the non-obese PCOS and control women; however, in the prior study reporting AD-related protein changes in PCOS, all the PCOS subjects had the metabolic phenotype A according to the Rotterdam criteria. PCOS phenotype A, that expresses all three of the diagnostic criteria, is reported to be at higher risk of adverse metabolic and cardiovascular outcomes compared to the other phenotypes, and phenotype D is the least severe [29]. In this study, all of the PCOS subjects had anovulatory infertility but half were phenotype B (irregular menses with hyperandrogenism) and half were phenotype C (irregular menses and polycystic ovaries on transvaginal scanning); there were too few subjects, and less than the power analysis would allow, to do a subgroup analysis. Therefore, the expression of AD-related proteins needs to be clarified for the individual PCOS phenotypes to determine if there is a potential increased risk only for the PCOS subjects with the type A phenotype.

It has been suggested that PCOS may have an increased risk of AD, with documented changes in cognition [16,18,19,20,21], and it is well recognized that patients with T2D have evidence of an increased risk for developing AD [10,11,12]; however, what this study shows is that, if the obesity and insulin resistance in these patients is addressed, that any AD-risk could possibly be normalized to that of matched controls. What is unknown is whether, once obesity and insulin resistance are established in PCOS, any intervention(s) to reverse these also positively impacts on the AD-risk proteins; however, this is inferred from the results that showed BMI correlated positively with APCS and apoE in PCOS women and that HOMA-IR correlated positively with apoE in PCOS women suggesting that, should weight and insulin resistance increase with an increased BMI, that these parameters would also increase. It has been suggested that underlying insulin resistance associated with PCOS is responsible for the alterations in cognitive function and, additionally, increases in inflammation [17]. Obesity is commonly a sequela of both PCOS and T2D, and obesity is also associated with an increased risk of AD [30]. This then leads to a complex milieu, with insulin resistance promoting increasing obesity due to compensatory hyperinsulinemia [31]; conversely, obesity, through mechanisms of chronic inflammation, adipokine activation, mitochondrial dysfunction [17], promotes insulin resistance. Thus, a vicious cycle may result. The inflammation that results from the insulin resistance/obesity may then be reflected in the development of cognitive impairment and the progression to AD [32,33]. In this study, those parameters associated with inflammation such as IL6 and the heat shock response proteins correlated with APP, SNCA and ApoE in the PCOS subjects but not the normal controls, suggesting that those with PCOS could be predisposed to enhanced changes of these proteins with the onset of inflammation induced by obesity and insulin resistance.

What role testosterone may have in the development of AD is debated but has been reported in men that a lower testosterone level was associated with AD [34]. Whether testosterone has a role in the development of AD in women is unclear, and in this study APCS negatively correlated with testosterone; however, this was the converse found in obese PCOS where APCS positively correlated; however, in both studies, testosterone levels did not correlate in the normal controls. Future studies to determine if testosterone in women has a positive or negative effect on AD risk need to be undertaken.

Intervention through recommended lifestyle management with 5–10% weight loss and undertaking regular physical exercise [35] does impact positively on obesity and insulin resistance [36], but is often difficult to sustain [37]; further, it is unknown whether AD-related risk factors are improved. Bariatric surgery has been shown to have a marked effect in PCOS, with reduction in BMI, insulin resistance, androgen levels and a return of regular menses, but no reports on AD-related risk factors are available [38]; however, the current evidence would suggest that early and sustained lifestyle changes may have a long term beneficial effect on cognition and AD-related risk and, at the very least, would not be harmful. Prospective studies on the effect of weight gain and weight loss and their effects on AD-related proteins in those women with PCOS would be highly informative.

A strength of this study is that it was performed on a homogeneous white Caucasian population though this would therefore need to be repeated to consider ethnic differences. The primary limitation of this study is that BMI was the only anthropometric analysis used and a more precise analysis of body composition, such as dual energy X-ray absorptiometry (DXA), resistance analysis, abdominal circumference or waist-to-hip ratio, would add value. In addition, the comparison between the protein levels between obese and the nonobese PCOS are limited, and the studies should be run using the same proteomic platforms; however, rigorous control samples included in every run would serve to mitigate this. Subsequent validation of the protein changes described with additional quantitative methods would also add value to the findings. Adjusting for BMI and insulin resistance is very difficult as both are so highly correlated with PCOS that regression adjustment for either or both would remove the PCOS effects; therefore, to determine if a decrease in AD-related risk factors is dependent on obesity and insulin resistance, this study provides the only design that would answer the question, with the caveat that it would have biased the PCOS phenotype recruited given that they all had to be non-obese.

## 4. Materials and Methods

### Study Design

In a cross-sectional analysis, plasma levels of Alzheimer’s-related proteins were measured in women with PCOS (*n* = 24) and control (*n* = 24) women recruited from the Hull IVF clinic [39]. Control women were age and BMI matched to the PCOS patients. All procedures performed in studies involving human participants were in accordance with the ethical standards of the Yorkshire and The Humber NRES ethical committee, UK, that provided approval for the study, and with the 1964 Helsinki declaration and its later amendments or comparable ethical standards. For the diagnosis of PCOS, the Rotterdam consensus criteria were used: (1) clinical (Ferriman-Gallwey score of >8) and biochemical hyperandrogenemia (a free androgen index (FAI) of >4) (2) oligomenorrhea or amenorrhea and (3) polycystic ovaries seen on transvaginal ultrasound [40]. Study participants had no other condition or illness and were required to be medication-free for nine months preceding study enrollment, including the exclusion of over-the-counter medication. Testing was undertaken to ensure that no patient had any of the following endocrine conditions: non-classical 21-hydroxylase deficiency, hyperprolactinemia, Cushing’s disease or an androgen-secreting tumor as per the recommendations [41]. Of note, both women with PCOS and control women had maintained a stable weight for at least 3 months prior to enrollment in the study. Demographic data for both control and PCOS women is shown in Table 1.

Patients presented after fasting overnight; height, weight and waist circumference and body mass index (BMI) were performed according to WHO guidelines [42]. BMI was defined as weight in kilograms and height in centimeters, with the formula kg/m^2^. Blood was withdrawn fasting and prepared by centrifugation at 3500× *g* for 15 min, aliquoted and stored at −80 °C. Analysis for sex hormone binding globulin (SHBG), insulin (DPC Immulite 200 analyser, Euro/DPC, Llanberis, UK), and plasma glucose (to calculate homeostasis model assessment-insulin resistance (HOMA-IR)) (Synchron LX20 analyser, Beckman-Coulter, High Wycombe, UK) was undertaken. Free androgen index (FAI) was derived from total testosterone divided by SHBG ×100. Insulin resistance (IR) was determined by HOMA-IR (insulin × glucose)/22.5). Serum testosterone was quantified using isotope-dilution liquid chromatography tandem mass spectrometry (LC-MS/MS) [39].

Plasma Alzheimer’s-related proteins were measured by the Slow Off-rate Modified Aptamer (SOMA)-scan platform (Somalogic, Boulder, CO, USA) [43]. Calibration was based on standards as previously described [44].

The protein quantification was performed using a Slow Off-rate Modified Aptamer (SOMAmer)–based protein array, as previously described [45,46]. Briefly, EDTA plasma samples were measured as follows (1) Analyte and primer beads binding-SOMAmers (fully synthetic fluorophore-labeled SOMAmer coupled to a biotin moiety through a photocleavable linker) were equilibrated; (2) Analyte/SOMAmers complex immobilization on streptavidin-substituted support. (3) Long-wave ultraviolet light cleavage to release analyte-SOMAmer complexes into the solution; (4) Analyte-SOMAmer complexes were immobilized on streptavidin support through analyte-borne biotinylation. (5) Elution of analyte-SOMAmer complexes with the released SOMAmers acting as surrogates for analyte quantification; (6) Quantification by hybridization to SOMAmer-complementary oligonucleotides. Normalization of raw intensities, hybridization, median signal and calibration signal were standardized for each [43,44].

Version 3.1 of the SOMAscan Assay was used, targeting the following proteins: Amyloid-precursor protein (APP), alpha-synuclein (SNCA), amyloid P-component (APCS), Pappalysin (PAPPA), Microtubule-associated protein tau (MAPT), apolipoprotein E (apoE), apoE2, apoE3, apoE4, Serum amyloid A (SAA), Noggin (NOG) and apoA1. In addition, proteins related to dementia were measured: fibronectin (FN), FN1.3, FN1.4, Von Willebrands factor (VWF) and extracellular matrix protein 1 (ECM1). Supplemental analysis of inflammatory proteins and protective heat shock proteins (HSPs) were determined that included Interleukin-6 (IL6), tumor necrosis factor-alpha (TNFa), HSP90AA1 (HSP90) and HSPD1 (HSP60).

## 5. Statistics

Power was based on APCS protein changes reported to be different in obese PCOS [14] (nQuery version 9, Statsol, Boston, MA, USA). APCS: for an alpha of 0.05 with an effect size of 0.9 then a total of 40 subjects (20 per arm) would be needed for 80% power if these proteins were to be significantly different in PCOS. Visual inspection of the data was undertaken followed by Student’s *t*-tests for normally distributed data and Mann-Whitney tests for non-normally distributed data as determined by the Kolmogorov-Smirnov Test. All analyses were performed using Graphpad Prism version 9.4.1 (San Diego, CA, USA).

## 6. Conclusions

PCOS patients who are non-obese and not insulin resistant show a lower AD-associated protein pattern risk profile that was no different to non-obese controls, indicating that lifestyle and interventional management to maintain optimal body weight may also be beneficial for the reduction of long-term risk for AD in PCOS.

## Figures and Tables

**Table 1:** Demographics, baseline, hormonal and metabolic parameters of the polycystic ovary syndrome (PCOS) subjects and controls (mean ± SD).

	Control (*n* = 24)	PCOS (*n* = 24)	*p*-Value
Age (years)	32.5 ± 4.1	31 ± 6.4	0.14
BMI (kg/m^2^)	24.8 ± 1.1	25.9 ± 1.8	0.56
Fasting glucose (nmol/L)	4.9 ± 0.4	4.7 ± 0.8	0.06
HbA1C (mmol/mol)	30.9 ± 6.5	31.8 ± 3.0	0.9
HOMA-IR	1.8 ± 1.0	1.9 ± 1.6	0.97
SHBG (nmol/L)	104.2 ± 80.3	71.7 ± 62.2	0.01
Free androgen index (FAI)	1.3 ± 0.5	4.2 ± 2.9	0.0001
CRP (mg L^−1^)	2.34 ± 2.34	2.77 ± 2.57	0.43
AMH (ng/mL)	24.3 ± 13.1	57.2 ± 14.2	0.0001

BMI—Body Mass Index; HbA1c—glycated hemoglobin; HOMA-IR—Homeostasis model of assessment—insulin resistance; CRP—C reactive protein; SHBG—sex hormone binding globulin; AMH—Anti-Müllerian hormone.

**Table 2:** Levels of Alzheimer’s-related proteins in non-obese women with polycystic ovary syndrome (*n* = 24; PCOS) versus controls (*n* = 24). Data presented are Mean ± 1 Standard Deviation of Relative Fluorescent Units (RFU).

	Control Mean (SD)	PCOS Mean (SD)	*p*-Value
APP	31,246 (19,569)	28,300 (22,332)	0.60
SNCA	9673 (3322)	10,012 (3343)	0.70
APCS	35,663 (11,717)	41,518 (11,879)	0.03
PAPPA	10,201 (5799)	10,065 (4505)	0.92
MAPT	129 (48)	130 (36)	0.89
apoE	25,296 (11,517)	26,588 (13,321)	0.69
apoE2	214,964 (67,686)	221,851 (56,020)	0.68
apoE3	148,321 (59,451)	157,453 (59,480)	0.56
apoE4	170,531 (57,851)	173,799 (63,642)	0.84
SAA	1211 (1150)	1585 (2774)	0.51
NOG	3081 (1063)	3203 (2582)	0.82
apoA1	12,344 (2248)	12,392 (2448)	0.94
vWF	11,481 (14,223)	10,746 (9306)	0.82
FN	18,553 (16,585)	19,991 (12,729)	0.71
FN1.3	4360 (7520)	3368 (2021)	0.50
FN1.4	63,653 (33,196)	69,376 (26,468)	0.47
ECM1	18,037 (6143)	19,345 (5284)	0.39

Amyloid-precursor protein (APP), alpha-synuclein (SNCA), amyloid P-component (APCS), Pappalysin (PAPPA), Microtubule-associated protein tau (MAPT), apolipoprotein E (apoE), apoE2, apoE3, apoE4, Serum amyloid A (SAA), Noggin and apoA1; von Willebrand factor (vWF); Fibronectin (FN); Fibronectin fragment 3 (FN1.3); Fibronectin fragment 4 (FN1.4); Extracellular matrix protein 1 (ECM1).

## Data Availability

All the data for this study will be made available upon reasonable request to the corresponding author.
